# Paracentesis in Acute Pleurisy

**Published:** 1852-02

**Authors:** 


*Paracentesis in Acute Pleurisy.* — In an article contained in the Medical Examiner, No. for Jan., 1852, entitled extracts from a lecture 'On the present position in Europe of some of the most interesting and important points of Modern Surgery,' recently delivered as an Introductory Discourse, by Thomas D. Mutter, M. D., Professor of Surgery in Jefferson Medical College, Philadelphia, we observe the following notice of paracentesis in Pleurisy: —

*Pleurisy.* — An operation altogether novel in the disease for which it was practiced, has been introduced by Professor Trousseau, of Paris, one of the most distinguished practitioners of that city of eminent medical men. Professor Trousseau told me that he has succeeded in relieving several patients who were almost in articulo mortis; and I have myself known it to accomplish the same end. The operation is nothing more than the evacuation of the fluid in cases of *acute pleurisy.* You are aware that the secretion is here often exceedingly rapid, and unless the lung be relieved, the patient must die of suffocation. When, therefore, you find a patient thus situated, recollect that paracentesis thoracis, promptly performed, will probably afford immediate relief.

That the rapid *secretion* of the fluid contained in the pleural sae in *acute pleurisy, often* exposes the patient to death by suffocation, is a novel fact. It is to be hoped that young practitioners who may discover the chest on one side to be filled with fluid, will not deem it necessary to perform the operation of paracentesis without a reasonable delay, notwithstanding this injunction to resort to the operation promptly, by so distinguished a surgeon.

Dr. Walshe, a pretty fair authority, says that in acute pleurisy, " Death is so rare a result of the disease when attacking individuals free from organic affections, that I have neither myself (and I have carefully attended to the point since my attention was first drawn to it, a year ago, by M. Louis) lost a patient from pure primary idiopathic pleurisy, with or without effusion, nor known of an occurrence of the kind in the practice of others." We may commend this statement to those who might be induced by Dr. Mutter's recommendation to puncture the thorax, as the reviewer of Dr. Walshe's treatise in the American Journal of Med. Sciences does to the " consideration of those who regard the disease as demanding bleeding, blistering, mercurials, and the whole armament of antiphlogistics."

The operation of paracentesis in cases of *chronic* pleurisy, in which the absorbing property of the membrane has been greatly impaired by the quantity of fibrinous exudation, and the degree of distension, has been proposed and practiced in this country within the past year, by a method simple and devoid of pain and danger. We allude to the plan originated by Dr. Morrill,

Wyman, of Cambridge, Mass., which was communicated to the profession by Dr. H. I. Bowditch, of Boston. Dr. Bowditch's article on this subject was copied into this Journal, and may be found in vol. VI. We observe in the Jan. No. of the American Journal of Med. Sciences, a report, by Dr. Bowditch, of several cases in which paracentesis with the small trochar attached to a suction apparatus was resorted to with marked advantage.

This procedure in the large accumulations of chronic pleurisy, certainly merits farther trial, but the proposition to tap the chest to remove the serum rapidly effused in cases of acute inflammation, is another matter, and, as it seems to us, should be protested against.

